# DONKEY: A Flexible and Accurate Algorithm for Clustering

**DOI:** 10.1021/acs.jctc.4c01750

**Published:** 2025-05-02

**Authors:** Jakub Kára, Kyle Acheson, Adam Kirrander

**Affiliations:** † Physical and Theoretical Chemistry Laboratory, Department of Chemistry, University of Oxford, South Parks Road, Oxford OX1 3QZ, United Kingdom; ‡ Department of Chemistry, University of Warwick, Coventry CV4 7AL, United Kingdom

## Abstract

We propose an accurate clustering algorithm suitable for the varied and multidimensional data sets that correspond to temporal snapshots from *on-the-fly* nonadiabatic trajectory-based simulations of photoexcited dynamics. The algorithm approximates the underlying probability density function using variable kernel density estimation, with local maxima corresponding to cluster centers. Each data point is then assigned to one of the maxima by employing a maximization procedure. Finally, clusters artificially separated by minor fluctuations in the probability density are merged. The algorithm does not require parameter tuning, which ensures flexibility and reduces the risk of bias. It is tested on several synthetic data sets, where it consistently outperforms conventional clustering algorithms. As a final example, the algorithm is applied to the excited dynamics of the norbornadiene ⇌ quadricyclane (C_7_H_8_) molecular photoswitch, demonstrating how distinct reaction pathways can be identified.

## Graphical abstract

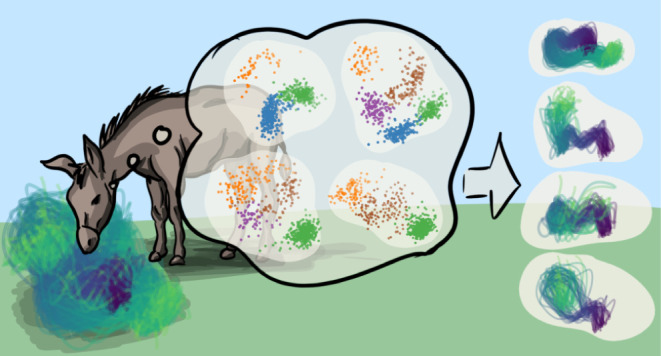

## 1 Introduction

Photochemical and photophysical processes are ubiquitous in nature.^1–4^ Studying the mechanisms behind the interaction of light and molecules deepens our understanding of this important class of microscopic processes and yields tangible real-world benefits, including organic light-emitting diodes (OLEDs), energy storage, and photovoltaic cells.^5–8^ Ultrafast pump–probe experiments^9–14^ which investigate photoinduced processes are generally accompanied by simulations to interpret the observations and better explain the underlying dynamics.

The exponential scaling of quantum mechanics means that the dynamics is often simulated using trajectory-based methods.^15–18^ Extracting insight from a large set of nonadiabatic trajectories is nontrivial and challenging by inspection alone. One route is dimensionality reduction via diffusion maps.^19,20^ An alternative approach for extracting information, particularly suited to trajectory-based simulations, is to group trajectories into mutually similar sets based on how similarly they evolve in space and time.^21–26^ Such clustering analysis makes it possible to identify representative trajectories corresponding to characteristic reaction pathways in the system. This reduces the number of trajectories that need to be manually inspected, since it is sufficient to inspect only a few representative trajectories for each cluster, and provides new insights about the photochemical reactivity of the system. It also automatically identifies the dominant modes of the dynamics. Furthermore, it offers the option to calculate computationally expensive observables, *e.g. ab initio* total X-ray scattering^27,28^ or photoelectron signals^29,30^ on a representative subset of the trajectories, reducing computational effort while increasing the accuracy of predictions. Clustering can thus be an effective method to extract maximum information from computationally expensive trajectories.

The ensemble of trajectories from a simulation approximates the molecular wavepacket and the data to be clustered is therefore quite distinct from other data sets where clustering is used routinely, such as bioinformatics,^31^ traffic,^32^ or networks.^33^ Following vertical excitation, the wavepacket (trajectory ensemble) initially forms a narrow distribution in phase space, which then rapidly evolves across coupled excited-state potential energy surfaces. Over the course of the dynamics, parts of the original wavepacket bifurcate and separate, where the size, dispersion, density, and shape of the different parts of the wavepacket may change significantly over time, which means that the characteristics of the data to be clustered also change. Despite the wide range of clustering algorithms available,^34,35^ few meet these exact requirements. Many are optimized for speed in very large and dense data sets and behave poorly when the size of the data set is small. Other algorithms fail to handle correctly the concavely shaped clusters which often appear in photoexcited dynamics when the wavepacket branches, while others require extensive fine-tuning of parameters to produce reasonable results, invariably leading to bias and a lack of generality.

It is also necessary that the algorithm does not require a prior on the number of clusters to be identified. As we shall explore later in this manuscript, these requirements are not met by existing clustering algorithms, with many of them requiring input parameters to be retuned in order to detect clusters that emerge with different sizes, densities, and widths at different time frames. Thus, this manuscript details the development of a new spatial clustering algorithm, DONKEY.[Fn fn1]. The proposed algorithm is specifically developed to deal with the aforementioned issues in spatial clustering of nonadiabatic dynamics trajectories over a range of independent time frames and does not require retuning of input parameters at different time frames. Our overall goal is to develop a flexible and general clustering algorithm for nonadiabatic dynamics by first addressing the problems with existing algorithms, benchmarking on suitable synthetic data sets, and providing a proof-of-concept application to nonadiabatic dynamics.

The paper is organized as follows: Section 2 covers the fundamental theory and the design of the algorithm; Section 3 evaluates its performance against conventional methods such as DBSCAN, OPTICS, and others,^36–39^ on synthetic data sets that challenge specific aspects of the algorithms; Section 4 demonstrates the algorithm’s performance for photoexcited processes by clustering trajectories that describe the dynamics in the norbornadiene ⇌ quadricyclane system. This molecular photoswitch has been the subject of recent experimental and theoretical studies^40–42^ and is being exploited for new energy-storage solutions.^7,43^ Toward the end of this section, we demonstrate the utility of DONKEY by manually identifying spatiotemporal clusters (channels) representing the dynamical evolution of distinct reaction pathways. Ultimately, having addressed the spatial clustering problem, we shall later look to combine DONKEY with temporal pattern mining algorithms^22^ for a fully automatic identification of spatiotemporal clusters.

## 2 Theory

### 2.1 Kernel Density Estimation

The central idea of the algorithm is to directly estimate the underlying probability density function (PDF) from which the data points are sampled. In our context, this density can be interpreted as the square norm of the nuclear wavepacket as well as related properties such as the electronic state populations, the momentum distribution, or predicted observables. We assume that every data point is obtained with some degree of uncertainty, which can be taken into account by convolving it with an envelope. Conventionally, the envelopes are referred to as kernels,^44^

$$K\left(x-Y_{i}\,\;\;|\;\;\;h\right)=f\left(\frac{\left|x-Y_{i}\right|}{h}\right)$$ 1

given by a real-valued symmetric and normalized function $f:R^{D}\to R_{0}^{+}$ evaluated at $x\in R^{D}$ with reference to the data point $Y_{i}\in R^{D}$ . The | ***x*** – ***Y***_*i*_| is the Euclidean distance, *h* a smoothing parameter, and *i* an index for the data points. We note that $R^{D}$ is the *D*-dimensional real vector space and $R_{0}^{+}$ is the set of non-negative real numbers. A kernel density estimation (KDE), *f̂*(***x***), for the PDF is then defined as the sum of the kernels that surround the *N* sampled data points, {***Y***_*i*_}, as follows,

$$\mathrm{f̂}\left(x\right)=\frac{1}{N}\overset{N}{\underset{i=1}{\sum }}K\left(x-Y_{i}\,\;\;|\;\;h\right)$$ 2

which is normalized and non-negative everywhere as required.

A common choice of kernel is a Gaussian distribution, *K* ^*G*^(***x*** – ***Y***_*i*_|*h*), selected for its analytically and numerically favorable properties,

$$K^{G}\left(x-Y_{i}\,\;\;|\;\;h\right)=\frac{1}{{\left(2\pi h^{2}\right)}^{D/2}}\exp\left[-\frac{1}{2}{\left(\frac{x-Y_{i}}{h}\right)}^{2}\right]$$ 3

where *h* is equivalent to the standard deviation. However, this is not the most general form in *D* > 1 dimensions; the standard deviation does not need to be identical in all dimensions and the coordinate axes need not align with the principal axes of the Gaussian. Thus, instead of a single *h*, the Gaussian kernel is defined using a symmetric positive-definite covariance matrix **C** such that,

$$K^{G}\left(x-Y_{i}\,\;\;|\;\;C\right)=\frac{1}{\sqrt{\det\left(2\pi C\right)}}\exp\left[-\frac{1}{2}{\left(x-Y_{i}\right)}^{T}C^{-1}\left(x-Y_{i}\right)\right]$$ 4

### 2.2 Covariance Optimization

KDE is an example of a nonparametric method for PDF estimation and although no assumptions are made about the PDF, *f̂*(***x***) will clearly depend on the choice of **C**.^45,46^ To obtain a suitable **C**, we use the unconstrained estimator of Leiva-Murillo and Artés-Rodríguez,^47^ which is designed to find a **C** that maximizes *L*(***Y***; **C**), the maximum *leave-one-out* likelihood of the KDE given by,

$$L={[\prod_{i}^{N}\mathrm{p̂}\left(Y_{i}\;\;|\;\;C\right)]}^{1/N}={[\prod_{i}^{N}\frac{1}{N-1}\sum_{j\neq i}^{N}K^{G}\left(Y_{i}-Y_{j}\,\;|\;C\right)]}^{1/N}$$ 5

where *p̂*(***Y***_*i*_ | **C**) is the maximum leave-one-out likelihood associated with point ***Y***_*i*_. For numerical evaluation, it is more convenient to consider $\log\left(L\right)=\frac{1}{N}\overset{N}{\underset{i}{\sum }}\log\left(\mathrm{p̂}\left(Y_{i}\,\;|\;C\right)\right)$ in the optimization instead. The leave-one-out prescription $\mathrm{p̂}\left(Y_{i}|C\right)=\frac{1}{N-1}\overset{N}{\underset{j\neq i}{\sum }}K^{G}\left(Y_{i}-Y_{j}\,\;|\;C\right)$ , which excludes ***Y***_*i*_ as a center from the sum, ensures that the optimization does not collapse all kernels into delta functions, which would correspond to a trivial solution of a plain likelihood maximization.^47,48^ By setting ∇_**C**_ *L* = 0, the following iterative procedure for calculating **C** is obtained,

$$C_{n+1}=\frac{1}{N}\times \underset{i}{\sum }\frac{\underset{i\neq j}{\sum }\left(Y_{i}-Y_{j}\right){\left(Y_{i}-Y_{j}\right)}^{T}K^{G}\left(Y_{i}-Y_{j}\,\;|C_{n}\right)}{\underset{i\neq j}{\sum }K^{G}\left(Y_{i}-Y_{j}\,\;\;|{\;\;C}_{n}\right)}$$ 6

One can thus iteratively optimize **C** by repeated substitution and evaluation until convergence is reached, **C**_*n*+1_ → **C**. We have found that overestimating the initial guess as an *N* × *N* identity matrix gives rise to a stable procedure, corresponding to finding the solution “from above”. This procedure is scale-independent, allowing us to conveniently normalize each feature to a range of [0,1]. A schematic illustration of the application of a KDE is shown in Figure 1a.

**Figure 1:**
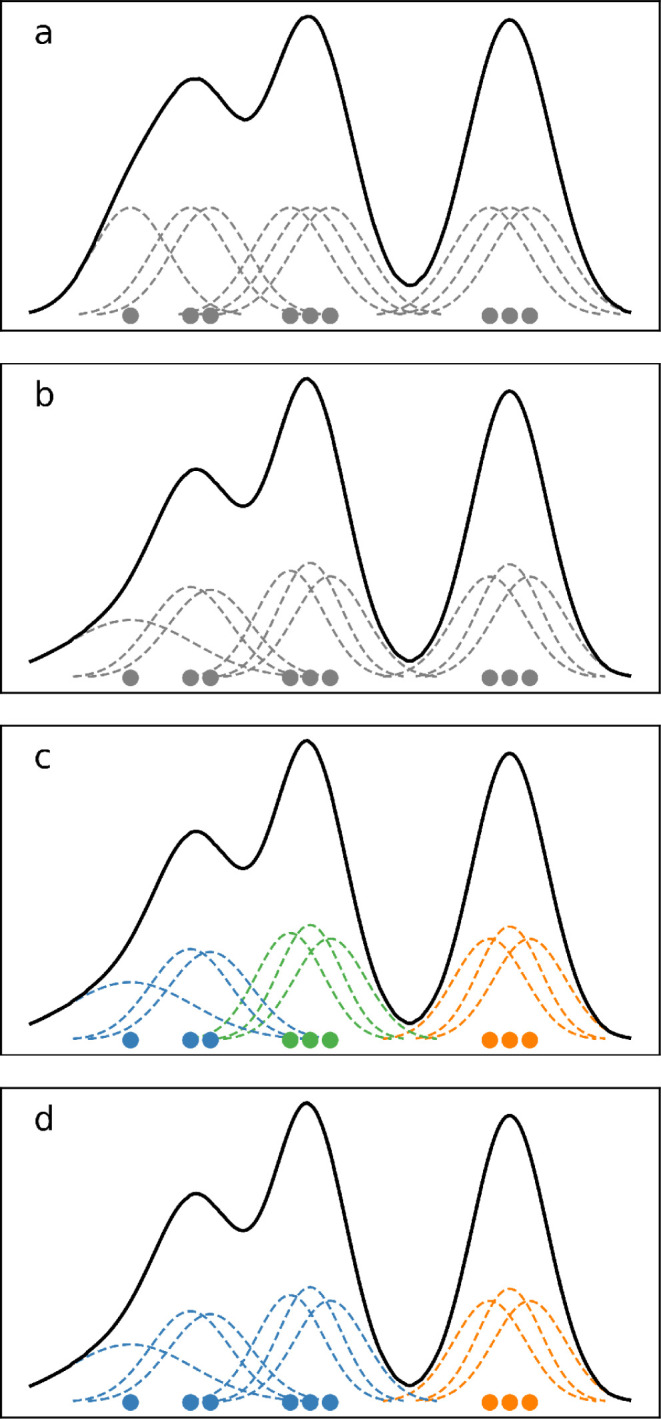
Schematic representation of the clustering steps in one dimension: (a) shows the KDE, (b) employs the local correction, (c) assigns points to KDE maxima, (d) merges clusters. Full points represent data points, dashed lines individual kernels, and full black line the total KDE. Colors reflect cluster assignments. Note that the effect of Abramson correction in (b)–(d) is exaggerated for illustrative purposes.

If infinitely many samples are drawn from a PDF, the distribution is reconstructed perfectly. However, we are limited to a small number of samples scattered throughout the feature space. In correspondence to the PDF, some regions exhibit higher density, while others are sparse. In other words, there is less uncertainty about the sampling in high-density regions than in low-density ones. We can reflect this observation by replacing the globally uniform pilot covariance matrix **C** obtained in the iterative procedure by a different covariance matrix **C** ^*k*^ at each data point *k*. We use the bias-minimizing procedure proposed by Abramson^49^ to locally rescale the pilot covariance,

7 $$C^{k}={\left(\frac{\mathrm{f̂}\left(Y_{k}\right)}{g}\right)}^{-1/2}C$$

with

8 $$g={\left(\underset{i}{\prod }\mathrm{f̂}\left(Y_{i}\right)\right)}^{1/N}$$

the geometric mean of {*f̂*(***Y***_*i*_)} calculated using the original uniform pilot covariance **C**. The kernel surrounding a data point ***Y***_*i*_ thus formally becomes *K* ^G^(***x*** – ***Y***_*i*_ | **C** ^*i*^), which renders the final KDE to the PDF,

$${\mathrm{f̂}}_{\mathrm{final}}\left(x\right)=\frac{1}{N}\overset{N}{\underset{i=1}{\sum }}K^{G}\left(x-Y_{i}\,\;\;|\;\;C^{i}\right)$$ 9

as depicted in Figure 1b, noting that in this example the adjustments of the width/height of the Gaussians are very minor.

### 2.3 Cluster Assignment

The local maxima of this PDF estimate, *f̂*_final_(***x***), can be interpreted as corresponding to the cluster centers. Data points are assigned to clusters by finding the dominant local maximum for each point through the means of density maximization. This constitutes a straightforward local optimization problem that benefits from the analytical derivatives of *f̂*_final_(***x***), making e.g., the Newton–Raphson algorithm efficient to implement. The assignment of data points to cluster centers yields a list of clusters, each associated with a subset of points, as shown in Figure 1c. However, such classification does not yield perfect results—similarly to how a ridge can link two peaks on the same mountain, two maxima in the KDE can be separated by a high-lying saddle point, even though they evidently should form a single cluster. To correct for this, a cluster merging step is carried out.

### 2.4 Cluster Merging

The merging procedure uses the information about the maxima and first-order saddle-points, determined from *f̂*_final_(***x***), to merge falsely separated clusters. The information is recorded in a merging matrix **M**, where the diagonal element *M*_*aa*_ is initially set to the PDF value at the center of cluster *a* and the off-diagonal *M*_*ab*_ to the PDF value at the saddle point connecting *a* and *b*. The diagonal elements have already been identified in the cluster assignment step above, and we proceed to locate the first-order saddle points between maxima. For this, we employ the algorithm by Smith,^50^ which transforms ${\mathrm{f̂}}_{\mathrm{final}}\left(x\right)\to \phi \left(x\right)$ , such that the maxima of ${\mathrm{f̂}}_{\mathrm{final}}\left(x\right)$ are mapped onto the first-order saddle points of ϕ­(***x***) and *vice versa*. Thus, finding the maxima of ϕ­(***x***) corresponds to locating the saddle points of *f̂*_final_(***x***). Maximizing ϕ­(***x***) is reasonably straightforward and does not require its explicit form, just the gradient and Hessian. First, find the eigendecomposition of **H**, the Hessian of *f̂*_final_: **U** ^T^ **HU** = **Λ** Second, transform ***g***, the gradient of *f̂*_final_, into the eigenbasis of **H**: ***g***_*p*_ = **U** ^T^ ***g***. Third, negate the lowest eigenvalue of **H** and the corresponding component of ***g***_*p*_, obtaining $\Lambda \to \Lambda^{\prime }$ and $g_{p}\to g_{p}^{\prime }$ . Finally, transform back to obtain $H^{\prime }={U\Lambda }^{\prime }U^{T}$ and $g^{\prime }=Ug_{p}^{\prime }$ , which can now be used in the optimization. To improve performance, the search for saddle-points is terminated if it ventures into a region where a third cluster carries greater weight in the KDE than the two clusters under consideration, or if the KDE decreases below a small threshold. In both cases, we conclude that the two clusters should not be merged directly, terminate the search, and set the off-diagonal *M*_*ab*_ = 0. The efficacy of locating saddle points is strongly dependent on the starting point for each local optimization; we set the starting point to the centroid of several pairs of closest points from each cluster, which usually converges within a few iterations. We can now formulate the following merging procedure:

(1) For each cluster pair (*a,b*), calculate $\mu_{ab}=\frac{M_{ab}}{\min\left(M_{aa},M_{bb}\right)}<1$ . This expresses the tendency of a lower-density cluster to be merged with a higher density one.
(2) Merge cluster pair (*a,b*) with the highest μ_*ab*_, provided that μ_*ab*_ > β, where the merging parameter β = exp(−1) reflects the drop-off in the Gaussian PDF one standard deviation from the center of the cluster. This ensures that the connecting ridge is sufficiently high to warrant merging. The merging procedure is terminated once no such pairs are found.
(3) Set *M*_*aa*_ = *M*_*bb*_ = max­(*M*_*aa*_, *M*_*bb*_) and *M*_*ab*_ = 0, then return to Step 2. This third step prevents a low-density cluster from causing two high-density clusters to be merged by indirectly linking them.

The clustering algorithm described above yields a final list of clusters commensurate with the approximate probability density, the KDE *f̂*_final_(***x***), as illustrated in Figure 1d. A complete workflow scheme of the algorithm is also provided in Figure 2. The specific value of β can in principle be varied, as discussed in Section 5. However, in the present work we keep the value of β fixed throughout for all the applications and data sets considered.

**Figure 2:**
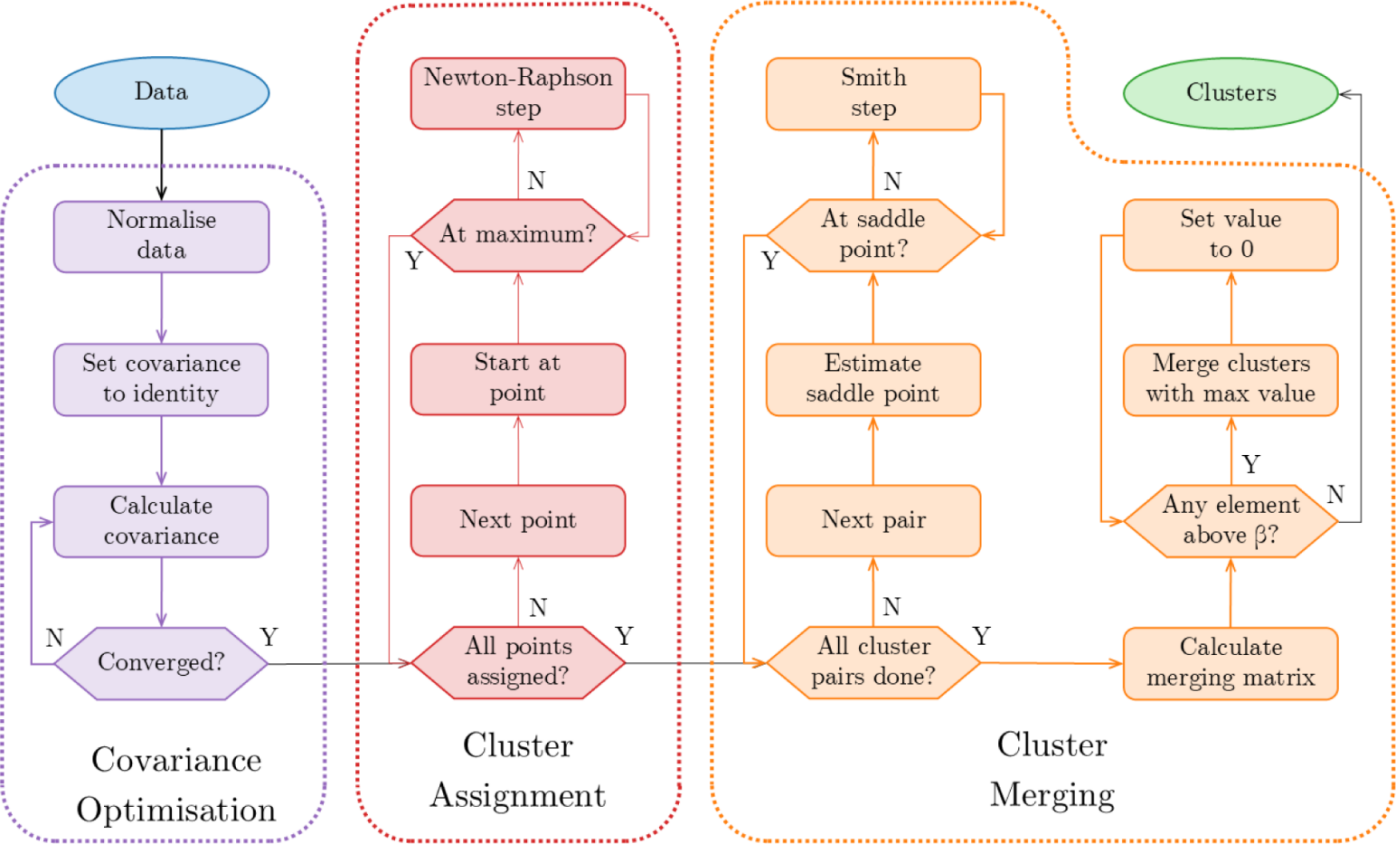
Schematic representation of the workflow in DONKEY.

## 3 Benchmarking

### 3.1 Evaluation Metrics

When benchmarking various clustering algorithms, it is helpful to have some metrics. We employ three metrics to quantify the quality of the clustering during the benchmarks: homogeneity, completeness, and V-measure. All three rely on knowing the ground-truth, which limits their use to benchmarks. In particular, they compare the class and cluster of each point, where classes constitute the correct separation of the data set according to the ground truth, i.e., “ground-truth clusters”. The metric of completeness measures the spread of the members of the same class among the identified clusters. Homogeneity, on the other hand, measures how spread out the members of the same cluster are among the classes that they originate from. As an illustration, let us consider two extreme cases. First, if all data points form a single cluster, the solution is perfectly complete (for every class, all members are in the same cluster) but entirely inhomogeneous (the members of the cluster originate from all classes). Second, if each data point forms its own cluster, the solution is perfectly homogeneous but entirely incomplete. The V-measure combines these two metrics by taking their harmonic mean and thus gives a balanced measure of the overall performance. All three scores are normalized to give results between 0 (the worst) and 1 (the best). Further details can be found in Ref.^51^

It must be noted that there is no objectively *best* metric to assess the results of clustering, a circumstance that fundamentally underpins the challenge of designing clustering algorithms. In the course of this work we have evaluated a number of alternative metrics, such as adjusted mutual information score^52^ or adjusted Rand index,^51^ which broadly yield results commensurate with V-measure. Further details are provided in the Supporting Information.

### 3.2 Two-Dimensional Synthetic Data

The benchmarking of our proposed algorithm, DONKEY, was performed on several synthetic data sets of varying sizes, topologies and dimensionalities. These are routinely used to evaluate the performance of clustering algorithms^53^ and the strong performance of DONKEY on these data sets is our main result. Each data set is generated from a known distribution representing the ground truth. In the first instance, we consider three synthetic two-dimensional sets, each offering a different challenge:

(1) *Blobs* – three same-variance Gaussian distributions, all showing high anisotropy;
(2) *Varied* – three Gaussian distribution of different variances, all overlapping;
(3) *Circles* – two separated concentric rings.

In the *Blobs* set, the challenge is to isolate clusters of high anisotropy, in *Varied* the algorithm must separate overlapping clusters and also contend with varying cluster density, and *Circles* constitutes an example of concave clusters. A benefit of the two-dimensional data sets is that the performance of the clustering algorithms can easily be assessed by inspection.

To evaluate the comparative performance of the new DONKEY algorithm, we benchmark it against established algorithms, including DBSCAN,^36^ HDBSCAN,^37^ OPTICS,^38^ Mean shift,^39^ and K-means,^54^ all of which are available in the *scikit-learn* package.^34^ To make the comparison as fair as possible, the parameters for each algorithm were tuned through an exhaustive parameter-space search to produce the best possible agreement with the ground truth, as judged by the V-measure score. The parameters can be found in the Supporting Information. Note that this type of parameter tuning, which is reliant on knowing the ground truth, overstates the performance of these algorithms compared to real-use cases.

Figure 3 shows 500 points data points generated for each of the three distributions (*Blobs*, *Varied*, and *Circles*) on a different row, with each column showing the results for a different clustering algorithm. The cluster assignments for each algorithm are color-coded (blue, orange, green, *etc*.). Additionally, (H)­DBSCAN and OPTICS can identify outliers, which are displayed in black. The performance of the clustering algorithms is also evaluated against the ground truth (i.e., the known distributions used to generate the clustered points) using the homogeneity, completeness, and V-measure metrics described in Section 3.1, with the results given in Table 1.

**Figure 3:**
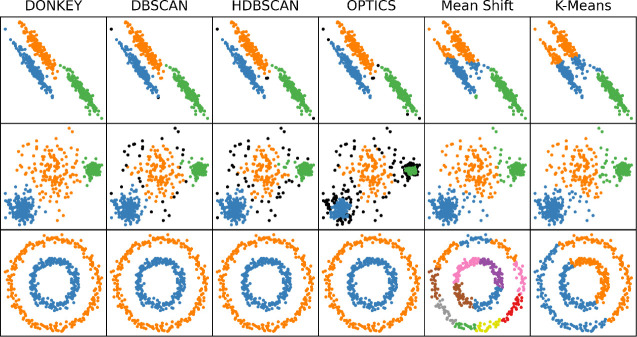
Results of clustering on the synthetic two-dimensional data sets with the selected algorithms. The top row shows the results for the *Blobs* data set, the middle row for the *Varied*, and the bottom row the *Circles*. The results for the different algorithms are shown in each column, as labeled, with the new DONKEY algorithm in the first column. The identified clusters are assigned different colors (blue, orange, green, magenta, *etc*), with outlier points in black (only for methods capable of flagging outliers, i.e., DBSCAN, HDBSCAN and OPTICS). Some colors are duplicated in *Circles*/mean shift due to the large number of clusters identified by the mean shift algorithm.

**Table 1:** Performance of the Clustering Algorithms on the Three Synthetic Two-Dimensional Datasets (*Blobs*,*Varied*, *Circles*) as Measured by Homogeneity (Top Row), Completeness (Middle Row), and V-Measure (Bottom Row)^a^

Data set	Metric	DONKEY	DBSCAN	HDBSCAN	OPTICS	Mean shift	K-means
*Blobs*	Homogeneity	**1.0000**	**1.0000**	0.9924	0.9862	0.6838	0.5803
	Completeness	**1.0000**	0.9807	0.9594	0.9366	0.6840	0.5808
	V-measure	**1.0000**	0.9902	0.9756	0.9608	0.6839	0.5806
*Varied*	Homogeneity	0.8607	**0.8628**	0.8565	0.6789	0.7810	0.7490
	Completeness	**0.8627**	0.7590	0.7338	0.5406	0.7901	0.7621
	V-measure	**0.8617**	0.8076	0.7904	0.6019	0.7856	0.7555
*Circles*	Homogeneity	**1.0000**	**1.0000**	**1.0000**	**1.0000**	**1.0000**	0.0000
	Completeness	**1.0000**	**1.0000**	**1.0000**	**1.0000**	0.2798	0.0000
	V-measure	**1.0000**	**1.0000**	**1.0000**	**1.0000**	0.4372	0.0000

a The best result(s) for each dataset is printed in bold.

We first consider the data set *Blobs*, shown in the top row of Figure 3, to examine the separation of clusters with high anisotropy not aligned with the feature axes. The results for DONKEY agree perfectly with the ground truth, yielding maximum scores across the board. (H)­DBSCAN and OPTICS are trailing closely behind, falsely identifying some outliers which lowers their completeness score. Both Mean shift and K-means are unable to separate the two close-lying clusters. Mean shift lacks the flexibility in its bandwidth parameter to describe the anisotropy properly, while the K-means partitioning algorithm cannot find the cluster boundaries, despite being given the correct number of clusters *a priori*. This example demonstrates the flexibility of DONKEY’s full covariance optimization for high anisotropy scenarios.

The second example, *Varied*, shown in the middle row of Figure 3, focuses on within-feature variance. DONKEY again achieves very reasonable separation with only minor discrepancies at the boundaries. (H)­DBSCAN performs similarly, only here the edges are often classified as outliers; DBSCAN’s homogeneity narrowly surpasses that of DONKEY. Mean shift and K-means manage to detect the general structure, but yield discrepancies around the edges of the denser clusters. Finally, OPTICS identifies many unnecessary outliers, making it the weakest performing algorithm in this scenario. The core problem for the other density-based methods considered here, notably DBSCAN and HDBSCAN, is that they use a global parameter in purely local decisions. In contrast, DONKEY addresses this with the already flexible KDE boosted by the local rescaling of the Gaussian covariances. The efficacy of this approach is highlighted by DONKEY achieving the highest V-measure score by a large margin, as seen in Table 1.

The third example, *Circles*, shown in the bottom row of Figure 3, challenges the clustering methods with concave shapes and a uniform, i.e., unpeaked, ground distribution along circular contours of constant radius. The result of K-means is, once again, completely unsatisfactory, for very similar reasons as in *Blobs*. Mean shift is able to separate the two rings, but only at the cost of severely fracturing their internal structure. The methods (H)­DBSCAN, OPTICS, and DONKEY all classify the two circles perfectly. This arguably represents the *a priori* most challenging scenario for DONKEY, since the estimated PDF is not smooth; the noise-introduced fluctuations in the uniform underlying distribution create many local maxima. The merging step is thus demanding and stretches the saddle point search to its limits, but *does* succeed in correct classification.

### 3.3 Multidimensional Synthetic Data

To complement the evaluation on two-dimensional data sets above, the clustering is examined on higher-dimensional data sets in the following. Each *D*-dimensional data set consists of *D* + 1 clusters (100 data points per cluster) sampled from a Gaussian distribution centered at a vertex of a regular unit *D*-simplex. Three scenarios are modeled by varying the standard deviation (σ) of the distributions: (i) no overlap with σ = 0.1, (ii) a small overlap σ = 0.2, and (iii) a large overlap with σ = 0.3. The results are shown in Table 2, following the same format as Table 1.

**Table 2:** Performance of the DONKEY Clustering Algorithm on Synthetic Multi-Dimensional Datasets for Different Values of the Standard Deviation *σ* of the Distributions^a^

Data set	Metric	*D* = 2	*D* = 3	*D* = 4	*D* = 5	*D* = 6	*D* = 7
σ = 0.1	Homogeneity	1.0000	1.0000	1.0000	1.0000	1.0000	1.0000
	Completeness	1.0000	1.0000	1.0000	1.0000	1.0000	0.9966
	V-measure	1.0000	1.0000	1.0000	1.0000	1.0000	0.9983
σ = 0.2	Homogeneity	1.0000	1.0000	1.0000	1.0000	0.8946	0.9899
	Completeness	1.0000	1.0000	1.0000	1.0000	0.9954	0.9866
	V-measure	1.0000	1.0000	1.0000	1.0000	0.9423	0.9882
σ = 0.3	Homogeneity	0.8980	0.9116	0.5142	0.7594	0.7709	0.4713
	Completeness	0.8981	0.9117	0.8813	0.8731	0.8629	0.7569
	V-measure	0.8980	0.9117	0.6495	0.8123	0.8143	0.5809

a The dataset with σ = 0.1 has no overlap, σ = 0.2 corresponds to a small overlap, and finally, σ = 0.3 to a large overlap. The clustering performance is measured using the metrics of homogeneity (top row), completeness (middle row), and V-measure (bottom row).

Clearly, DONKEY is able to separate the clusters reliably across all dimensions if the overlap is small. For large overlaps, it may struggle, as demonstrated in *D* = 4 and more strongly in *D* = 7, where many of the clusters merge (as borne out by the low homogeneity). The likely root cause here is the tail-heavy distribution in high-dimensional Gaussians, which bridges the gap between centers of high density. We note that this is an unfortunate feature of the data set, rather than a shortcoming of the algorithm, since the density-separation of the clusters is very small, resulting in multiple merges.

## 4 A Molecular Application

### 4.1 Trajectory Overview

Norbornadiene (NB) has received much attention as a molecular solar thermal (MOST) energy storage material.^55^ It can reversibly undergo a photochemical transformation to quadricyclane (QC) upon UV irradiation, storing solar energy in chemical bonds. This stored energy is released as heat upon reverting to norbornadiene via a catalytic reaction. The two isomers, depicted in Figure 4, are separated by a large potential barrier, since the conversion is a Woodward–Hoffmann forbidden [2 + 2]-cycloaddition (reversion) on the ground electronic state, S_0_. Photoexcitation to the first singlet excited state, S_1_, bypasses the barrier and undergoes efficient isomerization. The S_1_ → S_0_ decay proceeds via two symmetry-related conical intersections with rhombic molecular geometry, giving access to both ground-state isomers. The decay is rapid and occurs on a ∼ 50 fs time scale.^40,41,56,57^ Several representative potential energy cuts, which are motivated by the dynamics of the system, are available in the Supporting Information.

**Figure 4:**
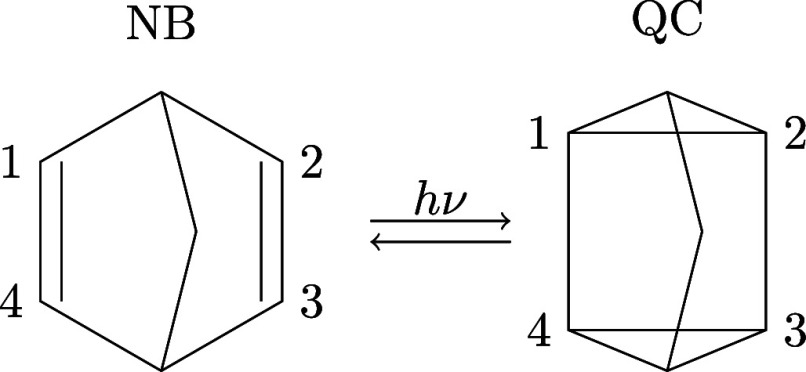
Schematic representation of norbornadiene (NB) and quadricyclane (QC) photoinduced isomerization, with key carbon atoms numbered. The chemical formula for the two isomers is C_7_H_8_.

Here, nonadiabatic semiclassical surface-hopping simulations of the photochemical dynamics in the NB/QC system are used as a typical example of photochemical dynamics, with a sparse time-series series of complex, multidimensional, and computationally expensive data points, which vary significantly in character and distribution across the time-series. For the current purpose of demonstrating a new clustering algorithm, the details of the dynamics are not particularly relevant, since the dynamics simply provides an archetypal time-ordered series of points in feature space with an underlying structure that we wish to extract.

For the simulations, an ensemble of 639 trajectories with initial conditions sampled from the Wigner distribution at the NB­(S_0_) equilibrium geometry is vertically excited onto the S_1_ electronic state. The trajectories are propagated independently using the fewest switches surface hopping algorithm for 80 fs with a stepsize of 0.5 fs, and time-derivative couplings were approximated through the norm-preserving interpolation from wave function overlaps.^58^ The electronic structure is calculated using state-averaged complete active space self-consistent field, SA(3)-CASSCF­(2,2), with a two-electron, two-orbital active space and a custom (trimmed) aug-cc-pvdz basis, as described in previous publications.^56,57^ The electronic structure is calculated using the Molpro package^59^ and the trajectories are calculated using our in-house dynamics software.

### 4.2 Preprocessing

Based on our previous work on the photochemistry of this molecular system, we identify three relevant degrees of freedom for the clustering as the wing separation, the rhombicity, and the wing length. These three degrees of freedom are specified in Table 3 and detailed analysis can be found e.g., in refs 40, 56, 57. For the initial feature space, we consider these three degrees of freedom, as listed in Table 3, and their respective rates of change, forming a six-dimensional data set.

**Table 3:** Key Coordinates Used as Features for the Clustering of NB ⇌ QC Trajectories^a^

Wing separation	$\frac{1}{2}\left[R_{12}+R_{34}\right]$
Rhombicity	[*R*_13_ – *R*_24_]
Wing length	$\frac{1}{2}\left[R_{14}+R_{23}\right]$

a The R_ab_ denote the distance between atoms C_a_–C_b_ using the atomic numbering in Figure 4.

The clustering is performed independently every 5 fs, starting in the Franck–Condon region, and following the evolution of the nuclear wavepacket throughout the dynamics. To reduce feature redundancy, principal component analysis (PCA) is performed on the six-dimensional data set. The reduced feature space consists of only those principal components, whose explained variance ratio exceeds 1%. The reduced space is recalculated for each clustering frame. Through this preprocessing, the computational cost is greatly reduced, while retaining the structure of the data set.

### 4.3 Spatial Clustering

A selection of results obtained with DONKEY is shown in Figure 5: until 30 fs, the nuclear wavepacket propagates along the wing separation coordinate, and only a single cluster is identified. Similarly, the cluster assignments from 70 fs onward remain constant. Therefore, we only show the clustering results in the temporal interval 30 ≤ *t* ≤ 70 fs, omitting earlier and later time frames.

**Figure 5:**
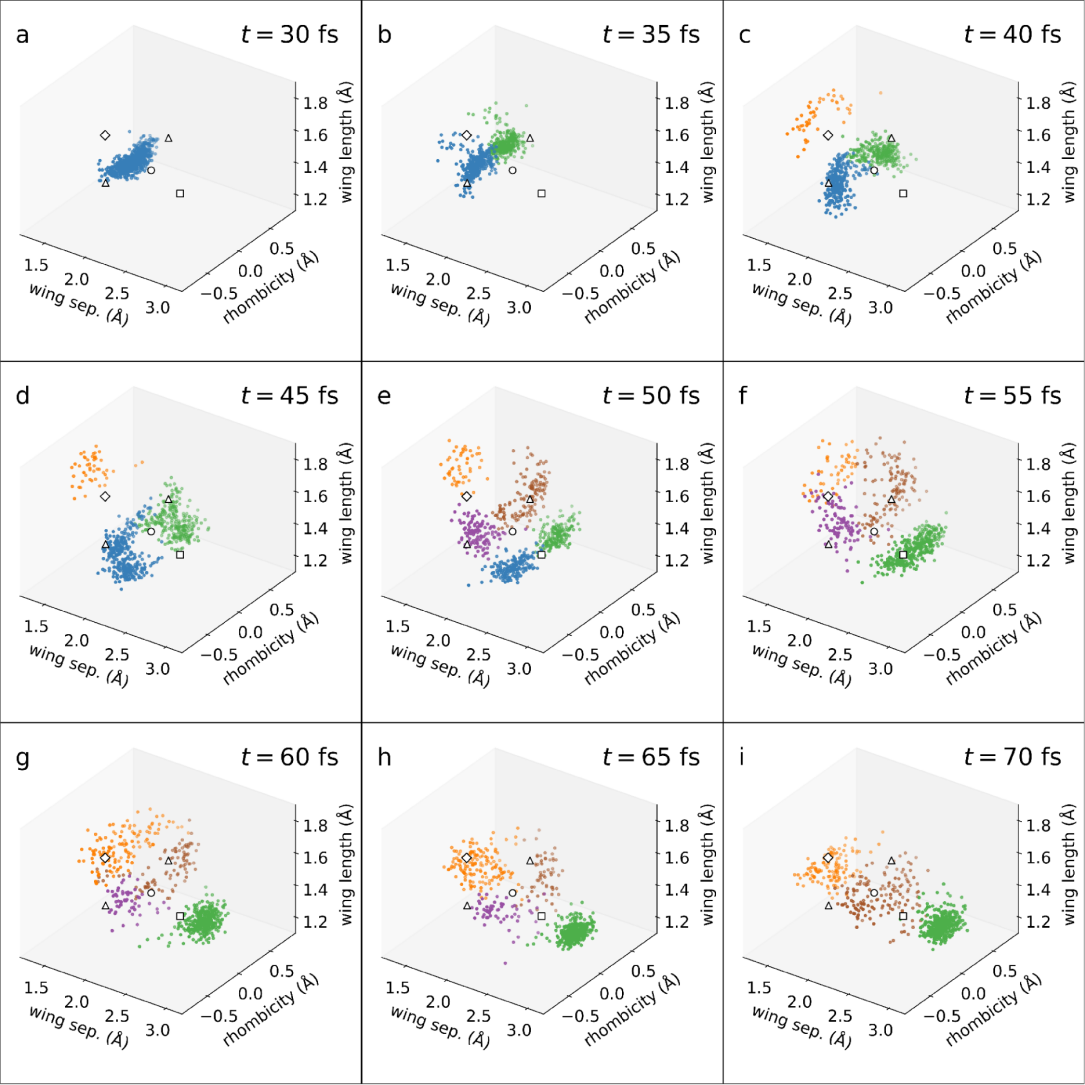
Clustering of NB/QC dynamics at selected times using DONKEY. The left-diagonal, right-diagonal, and vertical axes correspond to wing separation, rhombicity, and wing length, respectively, as defined in Table 3. Several important geometries are marked: NB and QC S_0_ minima (square and diamond), S_1_ minimum (circle), and the two symmetry-related minimum energy conical intersections (triangles). The timestamps (in fs) are shown in the upper right corner of each panel.

For contrast, Figure 6 displays the results of clustering the same snapshots with DBSCAN, which performs reasonably well on all of the benchmarks. Unlike in Section 3, however, no ground-truth is available here, making it much more difficult to optimize the tuning parameters. Instead, we employ a heuristic approach proposed by Sander et al.^60^ The MinPts parameter is set to 2*D*, where *D* is the dimensionality of the data set. Eps is determined by manually inspecting a *k*-nearest-neighbor (*k*NN) graph, where *k* = 2*D* – 1. Such a graph shows an “elbow” (sharp change in gradient), separating cluster points from noise; Eps is set to the *k*NN distance, where this occurs. This procedure is repeated for every frame independently.

**Figure 6:**
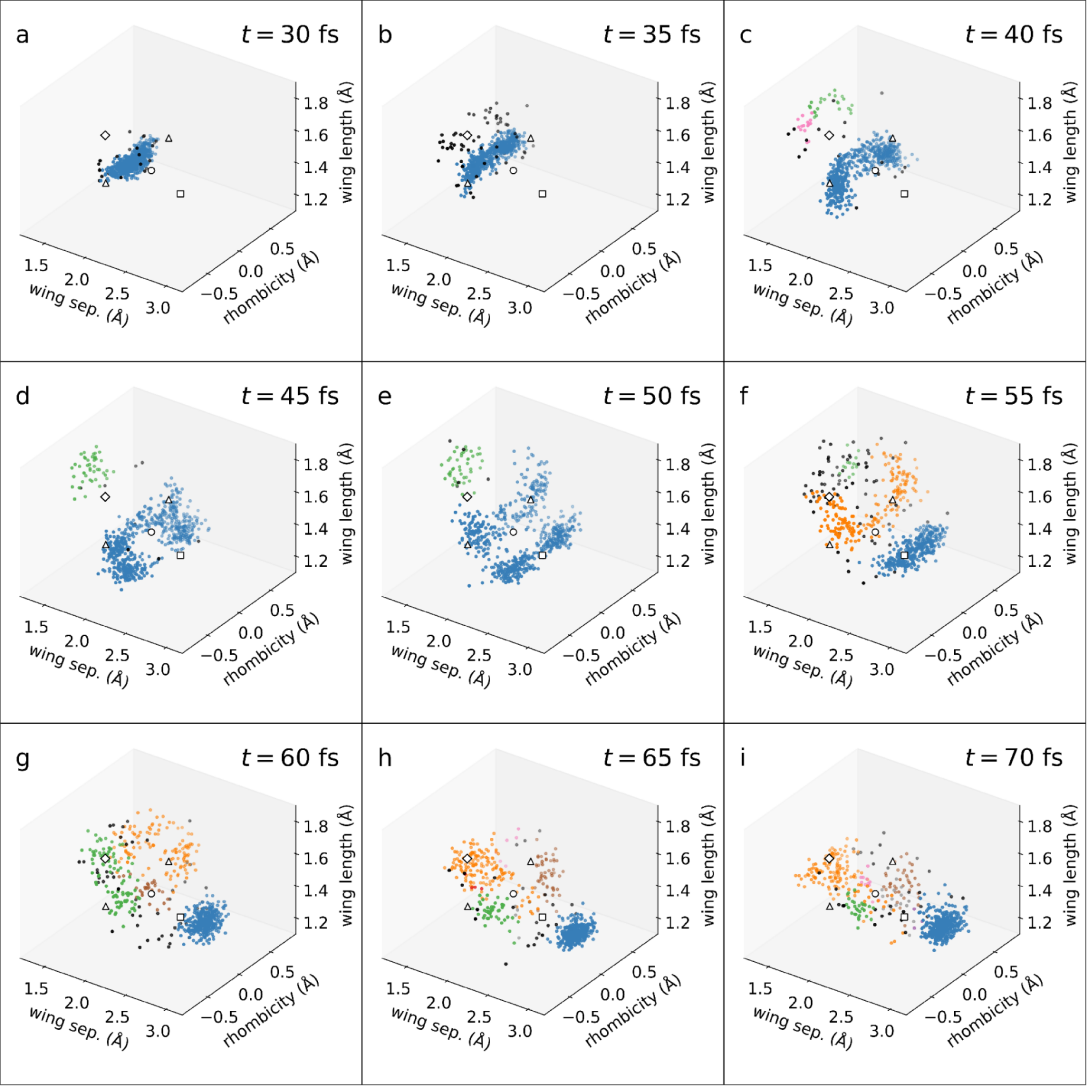
Clustering of NB/QC dynamics at selected times using the DBSCAN algorithm. The data considered and the axes and markers are identical to Figure 5. Note that the outliers which appear in the DBSCAN clustering are colored black. See Section 4.3 for a detailed discussion of the differences between Figures 5 and 6.

Comparing the results for the two clustering algorithms in Figures 5 and 6, stark differences can be spotted immediately. In the early dynamics (panels a–e), DONKEY shows a clear separation along the rhombicity axis, yielding two (or four) symmetry-related clusters. DBSCAN only identifies one cluster instead. We note that panel e, which is crucial for untangling and explaining the underlying dynamics, poses a great challenge—for DBSCAN to resolve the four clusters (akin to DONKEY) it would require Eps to be much lower, classifying the entire QC-like cluster as outliers. In the later stages (panels g–i), a similar scenario to the *Varied* data set is encountered, where DBSCAN cannot resolve clusters of varying densities. Overall, this comparison illustrates how the flexibility of DONKEY leads to higher-quality results, without any need to tinker with clustering parameters.

### 4.4 Temporal Clusters

Next, we connect the clusters obtained by DONKEY at each individual time frame into a set of continuous spatiotemporal clusters^22^ by identifying trajectories that exhibit similar behavior, i.e., cluster identically over some chosen “checkpoint” frames. Here we pick *t* = 40, 50, 70 fs, which capture the initial bifurcation, the decay through CIs, and the final distribution of products. As such, this is essentially equivalent to manual inspection followed by sorting by frame by frame. Figure 7 shows the reaction channels obtained by this procedure, which collectively capture the temporal evolution of 598 out of the 639 trajectories.

**Figure 7:**
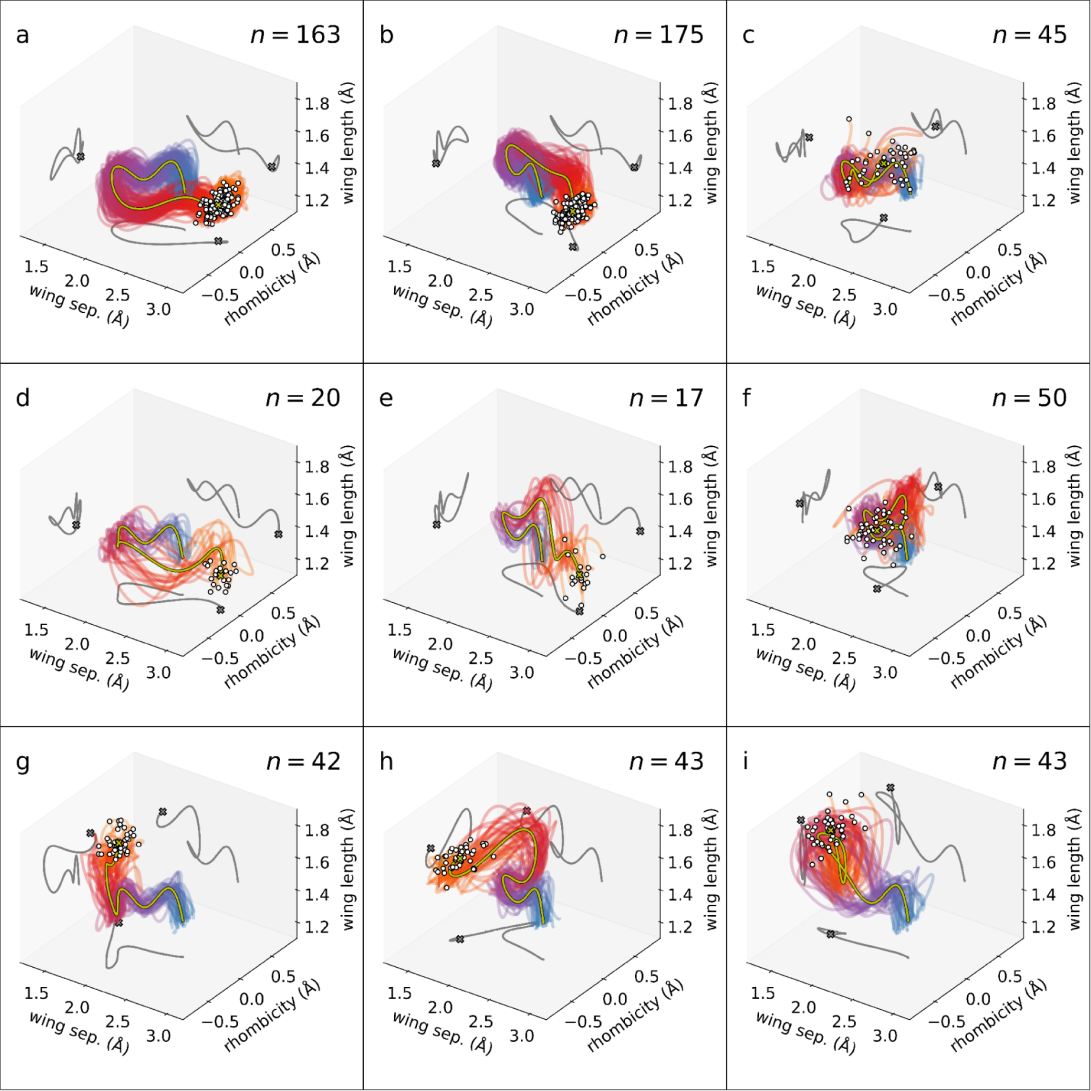
Relaxation channels for the NB/QC dynamics identified using clustering. Each panel shows a different cluster, with the total number of trajectories in each cluster shown at the top right corner of each panel. The time axis for the trajectories is indicated by the color along the trace of each trajectory—blue corresponds to *t* = 0 fs, while orange marks *t* = 80 fs, with the final configuration highlighted by a white circle. The yellow curve depicts a representative (centroid) trajectory of the set with its final configuration marked a cross and its projection onto all axis–axis planes shown in gray. The trajectories originate at the NB equilibrium geometry. The coordinate axes are identical to Figure 5.

Until this point, we have only considered nuclear information (positions and velocities). Of course, this is only half the story in nonadiabatic processes, since the electronic states play a crucial role. However, the electronic subsystem clearly influences the nuclear dynamics. For instance, in surface hopping, the active surface is stochastically decided by the quantum populations and their flux. Two trajectories evolving on different surfaces will have distinct time evolution and should eventually separate. We proceed to correlate the current clustering with the information about electronic state populations known from the simulations. First considering the clusters, we can tentatively assign the final state of each reaction with the aid of the key geometries included in Figure 5. This gives that the pathways a, b, d, e are ground-state NB, that g, h, i are ground-state QC, and that c, f remain on the excited state. We compare this to the actual average quantum populations as calculated from the simulations for each channel, as shown in Figure 8 together with their standard deviations. Encouragingly, the electronic state population dynamics can be seen to be quite homogeneous for each identified cluster, with the trajectories in the cluster following similar patterns of nonadiabatic transfer, as expected. In the Supporting Information, a version of Figure 8 is included which also shows when the surface hops occur for each cluster.

**Figure 8:**
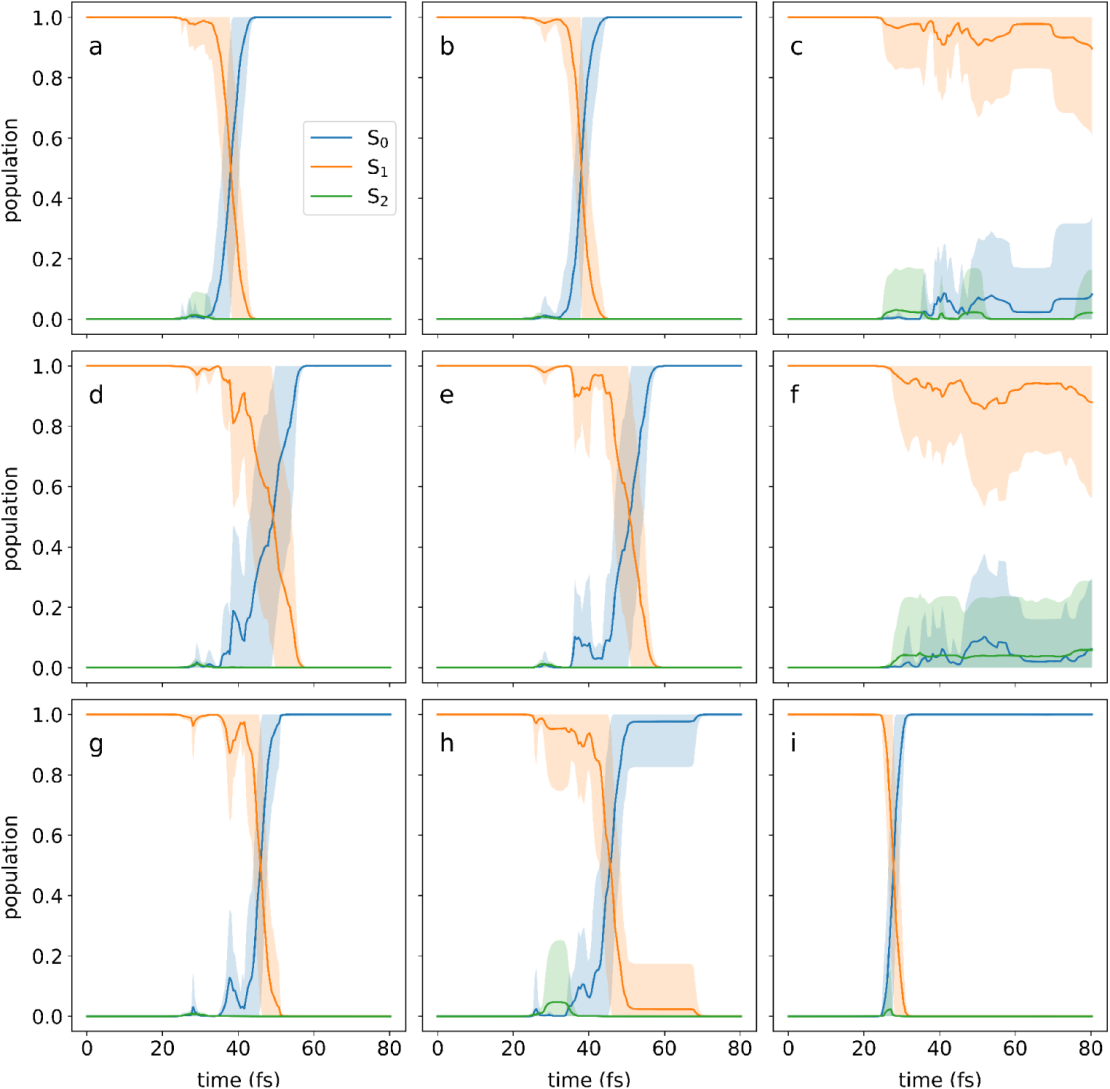
Populations on the electronic states S_0_, S_1_, and S_2_ for each of the relaxation channels in Figure 7, as calculated from the simulations. The full lines correspond to the average quantum population calculated for each pathway with the shading corresponding to one standard deviation.

Now, analyzing the dynamics of the trajectories in the different clusters, most of the trajectories hit a barrier on the S_1_ potential energy surface at *t* = 35 fs, which splits the wavepacket symmetrically along the rhombicity internal coordinate. The trajectories then travel parallel to the barrier into a region of high nonadiabatic coupling, where many relax to the ground state. On the S_0_ ground state, another bifurcation occurs—some trajectories proceed to QC-like geometries (subplots g, h in Figure 7), others return to NB-like geometries (subplots a, b, d, e), and the rest stay on the S_1_ excited state (c, f), oscillating around the conical intersection. Several trajectories (22 in total; not included in Figure 7) oscillate around the S_1_ minimum without ever reaching either of the conical intersections; in the static picture, these are indistinguishable from those returning from CI regions (c, f), as can be seen in Figure 5i. Finally, after the initial bifurcation on the S_1_ state, some trajectories continue to decrease their wing separation (subplot i) corresponding to a ballistic NB → QC conversion followed by rapid decay to the S_0_ ground state. We note that 19 trajectories are not classifiable under this scheme and represent either very unlikely pathways or outliers.

Interestingly, inspection of the trajectories included in the two spatiotemporal clusters shown in the subplots a and d (as well as their symmetrical counterparts b and e) have the same distribution, yet are identified as different pathways. This is due to the fact that while they undergo nearly identical geometric changes, i.e., they are spatially similar, their *temporal* evolution is different. Specifically, the trajectories in subplots d and e are delayed since they spend more time oscillating around the conical intersections. This is most visible in Figure 5 when looking at the time-frames between times 40 and 55 fs. The clustering thus succeeds to separate trajectories not only based on differences in their spatial, but also in their temporal evolution.

## 5 Discussion

A key point to note, is that all the clustering results shown for the DONKEY algorithm in this paper were achieved without any parameter tuning whatsoever; once the features were selected, no further user intervention was required. This is especially important for time-dependent studies, where any parameters would normally have to be (re)­tuned for every time frame, introducing inconsistency and bias. This is in stark contrast with many of the established clustering algorithms, such as those used for benchmarking in Section 3, where often multiple sensitive parameters had to be carefully selected to obtain reasonable results. Despite this great simplification for DONKEY, as shown throughout this study, the final results often surpass the best possible results with the other algorithms. This is clearly a significant strength of the DONKEY algorithm and this flexibility makes it eminently suitable for clustering photochemical dynamics. Nevertheless, in anticipation of even more challenging scenarios and applications in the future, we propose three parameters that *could* be tuned, should the need arise.

The global broadening parameter α is used to adjust the Gaussian widths uniformly in all dimensions, such that

10 $$\begin{array}{l} K\left(x-Y_{0}|C,\alpha \right)=\sqrt{\frac{\alpha }{\det\left(2\pi C\right)}}\times \\ \times \exp\left[-\frac{1}{2}\alpha {\left(x-Y_{0}\right)}^{T}C^{-1}\left(x-Y_{0}\right)\right] \end{array}$$

where α = 1 by default. This should be applied when the estimated covariance exhibits over- or under-smoothing, in which case α should be increased or decreased, respectively. Changing α does not require rerunning the whole algorithm from scratch; the optimized covariance matrix for a given data set is independent of α and the algorithm can thus be restarted just before the local maxima search. Since the optimization procedure is often the most computationally intensive step, this reduces the costs of tuning α to a fraction of the total algorithm runtime.

The merging parameter β is used to increase or decrease the merging threshold for artificially separated clusters, as discussed in Section 2. In particular, we require μ_*ab*_ > β for at least one cluster pair (*a,b*) to continue the merging. By default, β = 1/e. Tuning β, should this be necessary, comes at virtually no cost, as it is performed after both the maxima and the saddle point searches.

An additional outlier parameter γ can be specified to detect outliers in the data set, if such classification is required. If ***Y***_0_ initially belongs in cluster *c*, then ***Y***_0_ is classified as an outlier if *f̂*_final_(***Y***_0_)/*M*_*cc*_ < γ, where *M*_*cc*_ is the value of *f̂*_final_ at the center of *c*. By default, outlier identification is not used, thus γ = 0. Changes to γ can be made at no extra cost, since all the required values were already calculated previously. Examples illustrating the use and sensitivity of all three parameters, α, β, and γ, can be found in the Supporting Information. Briefly, both α and β are not sensitive to decreasing their values, but result in fracturing of the clusters if they are made larger; γ, on the other hand, only acts as a noise filter, and does not affect the performance.

Finally, the Abramson correction for the local bandwidth estimation may be disabled. This could be done when one suspects that there may exist some underlying structure within the tails of the probability distribution, since the local broadening coefficients will smooth out any such features.

It is worth re-emphasizing that the potential optimizations of the algorithm proposed above constitute parameters that would only be tuned once. This stands in stark contrast to the tuning required with standard clustering algorithms, which have to be retuned at each different time frame to cope with the changing characteristics of the data over the course of photochemical dynamics.

We also note that one may consider clustering on other types of features than the distance-based features considered here, for instance torsional or dihedral angles. In such cases, extra care is required to account for the 2π periodicity of such angular coordinates. An extension to periodic features can intuitively be incorporated in the present method by employing periodic boundary conditions (PBCs) and then evaluating the Gaussians using the minimal image convention. Because of the exponential nature of Gaussians, the PBCs will only become relevant near the periodic boundaries and will have little-to-no effect near the center of the feature space. In cases where the original feature space is transformed, for instance using PCA, the boundaries are no longer parallel to the coordinate axes and a more careful treatment is needed to identify the minimal image. We suggest finding such images in the original feature space, but then carrying out the actual calculations in the transformed (reduced) space. This idea has not yet been fully implemented, and future investigations on other molecules will indicate how practical it is.

Finally, a few words on the computational scaling of the algorithm. Given the explicit estimation of the underlying PDF and the extremum searches, the computational cost of DONKEY is high compared to conventional clustering methods. In particular, the time complexity of the covariance matrix optimization is $O\left(N^{2}D^{3}\right)$ , where the term *N* ^2^ comes from the double sum over trajectories in eq 6 and *D* ^3^ from the necessity of calculating **C** ^–1^. If a naïve Newton–Raphson method is used to locate the maxima, the time complexity of this step is $O\left(N^{4}D^{3}\right)$ , where *N* appears in the sum in *f̂*, *N* ^2^ in the evaluation of the Hessian matrix elements, and *D* ^3^ in the Hessian inverse, and this operation has to be repeated for each of the *N* data points. Finally, the saddle point search yields $O\left(N^{3}M^{2}D^{3}\right)$ , which is identical to the previous step with *N* data points replaced by $O\left(M^{2}\right)$ cluster pairs, where *M* is the number of clusters. In practice, the prefactor associated with the first step is much larger than for the other two, which makes it competitively expensive for *N* ≤ 1000. Additionally, only neighboring clusters contribute to the saddle point search, making this step by far the cheapest.

## 6 Conclusions

This study presents a new clustering algorithm (DONKEY) developed for comparatively small data sets where accuracy and flexibility are a priority. Using an estimation of the underlying probability density function, the algorithm is able to consistently identify clusters in challenging synthetic data sets. Crucially, no specific user inputs or parameter-tunings are required to perform clustering across the entire varied time-series represented by trajectories from nonadiabatic simulations. This sets DONKEY apart from other methods as an ideal candidate for fully automated classification of data sets that change character over time, as typical for photochemical dynamics.

The utility of the algorithm is demonstrated by applying it to an ensemble of trajectories corresponding to the dynamics of photoexcited norbornadiene, allowing us to identify key reaction pathways. The excellent performance of DONKEY is underscored by comparison to DBSCAN, an existing clustering algorithm that performed well when tested on the synthetic data. It is conceivable that DBSCAN could perform as well or even better than DONKEY in some limited scenarios, such as a very ballistic process with little dispersion or branching, but even in those scenarios we anticipate that DONKEY will perform well while being more general and flexible. Notably, when applied to the trajectory data set considered in this paper, DBSCAN failed to make sensible cluster assignments.

The clustering assignments by DONKEY were further appraised with respect to the electronic population dynamics, known from the simulations. Despite that this information was not explicitly included in the clustering, the trajectories in each channel exhibited distinct and uniform population dynamics. This validates clustering as a sensitive tool for analysis, complementary to e.g. population analysis and the identification of key features on the potential energy surfaces (energy minima, barriers, minimum energy conical intersections and crossing points, *etc*).^61^ Finally, in anticipation of even more challenging clustering problems that may be encountered in future applications, in Section 5 we present potential performance improvements and adjustments, and outline how periodic features could be accounted for.

In terms of future work, there are two main directions. The most important is an automated algorithm for temporal pattern mining. While DONKEY is effective for the clustering of individual time frames, as illustrated for NB/QC, identifying the spatiotemporal clusters (i.e. channels) for the NB/QC dynamics was done in a partially manual fashion. Fully automatic temporal pattern mining from a database of spatial clusters has specific challenges, mainly relating to how trajectories bifurcate and how trajectories can leave and reenter clusters. We aim to develop a flexible method for temporal pattern mining, that utilizes the efficient spatial clustering by DONKEY to yield an overall automatic and robust approach to spatiotemporal cluster identification.

Another important area to consider is the selection of features for clustering. This includes evaluating how the algorithm performs in complex systems with a large number of degrees of freedom, a more careful investigation of periodic features, and the inclusion in the clustering of features that do not explicitly relate to nuclear motions, such as electronic state populations. Finally, there are other interesting directions to explore further, such as exploitation of clustering when running trajectory-based methods that involve coupling between different trajectories.^62,63^ It should further be noted that clustering methods may be quite useful when solving the inverse problem in a trajectory basis, aiming to identify the trajectories that best explain the observed experimental signal.^64^ Given the density-based character of DONKEY, it would also be intriguing to explore how it might be adapted for the analysis of numerical grid-based quantum dynamics simulations.^20^

In summary, the current work introduces DONKEY as a powerful, flexible, accurate, and essentially parameter-free clustering method highly suitable for automatic clustering of trajectories from nonadiabatic simulations. The algorithm outperforms established methods in challenging data sets and could potentially be applied in demanding circumstances unrelated to quantum dynamics, such as advanced image analysis or complex multidimensional data sets from experiments.

## Supplementary Material



## Footnotes

a Density Optimisation with Normal Kernels to Estimate Yields.

## Notes

All parameters for other algorithms used in benchmarks; runtimes for benchmarks; PESs for the QC/NB dynamics and a version of Figure 8 which includes information on when hopping occurs; runtimes on the QC/NB data set; discussion of the sensitivity of α, β, and γ (PDF)
