# Supplementary material for: DONKEY: A Flexible and Accurate Algorithm for Clustering
Source: J Chem Theory Comput. 2025 May 2;21(12):5789–802. doi: 10.1021/acs.jctc.4c01750 (PMC12199456; doi:10.1021/acs.jctc.4c01750)
Supplement: Supplementary file 1 [file ct4c01750_si_001.pdf]

Supporting Information for DONKEY: a flexible and accurate algorithm for clustering

Jakub Kára,*,† Kyle Acheson,‡ and Adam Kirrander*,†

†*Physical and Theoretical Chemistry Laboratory, Department of Chemistry, University of Oxford, South Parks Road, OX1 3QZ Oxford, United Kingdom*

‡*Department of Chemistry, University of Warwick, Coventry CV4 7AL, United Kingdom*

E-mail: Jakub.Kara@lincoln.ox.ac.uk; Adam.Kirrander@chem.ox.ac.uk

1 Benchmarking

Below are listed all the necessary parameters to replicate the best-performing configurations for each algorithm-dataset pair the main text. The names of parameters directly correspond to the keywords used in the scikit-learn package.[1]

1. *Blobs*

   DBSCAN: eps = 0.26, minPts = 5

   HDBSCAN: min_samples = 2, min_cluster_size = 9

   OPTICS: xi = 0.12, min_samples = 8, min_cluster_size = 0.2

   Mean shift: bandwidth = 0.58

   K-means: n_clusters = 3

2. *Varied*

   DBSCAN: eps = 0.267, min_samples = 6

   HDBSCAN: min_samples = 4, min_cluster_size = 7

   OPTICS: xi = 0.01, min_samples = 7, min_cluster_size = 0.2

   Mean shift: bandwidth = 0.74

   K-means: n_clusters = 3

3. *Circles*

   DBSCAN: eps = 0.3, min_samples = 5

   HDBSCAN: min_samples = 3, min_cluster_size = 15

   OPTICS: min_samples = 7, xi = 0.08, min_cluster_size = 0.1

   Mean shift: bandwidth = 0.68

   K-means: n_clusters = 3

Table S1: Performance of the clustering algorithms on the synthetic datasets as measured by the adjusted Rand index. The best result for each dataset is printed in bold.

Dataset	DONKEY	DBSCAN	HDBSCAN	OPTICS	Mean shift	K-Means
Blobs	**1.0000**	0.9940	0.9879	0.9788	0.7027	0.5467
Varied	**0.8906**	0.8243	0.8031	0.5184	0.7968	0.7585
Circles	**1.0000**	**1.0000**	**1.0000**	**1.0000**	0.1795	-0.0019

Given the ground truth, an alternative way of assessing the performance of a clustering algorithm is the adjusted Rand index (ARI),[2]. It is based on the Rand index (RI)[3] which measures the similarity between two label assignments and returns a value between 0 and 1, with 1 being absolute agreement.[3] In the case of benchmarks using synthetic data, the distribution used to generate the data is known and the labels obtained by each algorithm can be compared to the ground truth. However, even if the classification was random, some labels would still match. The expected coincidental agreement forms a baseline, which is subtracted from the RI to yield the ARI.[2] Consequently, ARI can yield negative values, which can be interpreted as "worse than random" while the upper bound remains 1.

Table S2: Runtime of the clustering algorithms on the synthetic datasets measured in seconds.

Dataset	DONKEY	DBSCAN	HDBSCAN	OPTICS	Mean shift	K-Means
Blobs	2.148	0.005	0.006	0.397	0.069	0.112
Varied	1.847	0.004	0.005	0.359	0.104	0.045
Circles	2.271	0.004	0.007	0.342	0.088	0.088

As mentioned in the main text, running DONKEY can be quite expensive – a price to pay for the accuracy and flexibility. Table S2 gives runtimes for all the synthetic datasets on a local machine. Note that these values correspond to a single run, and therefore do not represent the total effort to perform the clustering. In order to tune the parameters (listed above), a parameter-space search has to be conducted and the results compared to some objective metric (ground truth in this case), which may increase the total wall time by one or two orders of magnitude. Furthermore, all of the conventional algorithms, unlike DONKEY, are heavily optimised, increasing the difference even more.

2 Dynamics

Table S3: Runtime of the DONKEY on norbornadiene dynamics measure in seconds. The Total time (last column) is broken down into the three major components: covariance optimisation, maxima detection, and merging.

Frame	Covariance	Maxima	Merging	Total
30 fs	7.87	1.26	0.05	9.33
35 fs	7.73	1.32	0.16	9.35
40 fs	2.36	1.01	0.18	3.67
45 fs	8.37	1.26	0.30	10.07
50 fs	7.23	1.31	0.30	8.98
55 fs	4.11	1.35	0.50	6.10
60 fs	3.22	1.36	0.40	5.11
65 fs	3.13	0.83	0.16	4.23
70 fs	3.48	0.87	0.26	4.72

Table S3 shows the runtime and its components for NB/QC dynamics clustering for the frames shown in the main text.

3 Parameter Sensitivity

The main text proposed three possible tuning parameters that could be used to improve the clustering results in some cases. Here we show their effect on the 2D benchmarks.

Figure S3 shows the effect of changing α, the broadening parameter – increasing α compresses the distribution and *vice versa*. In the case of broadening down to $\alpha = 0.5$, the results are essentially identical to the default values; compressing can cause more clusters to be identified, as the kernels are more local. The quality of clustering is quantified in Table S4 via the familiar homogeneity, completeness, and V-measure.

The effect of changing the merging threshold, β, is demonstrated in Fig. S4. Again, the results are mostly similar to the default case, when moving towards the lower β, but increasing the parameter may lead to fracturing the clusters and suboptimal performance, as quantified in Table S5.

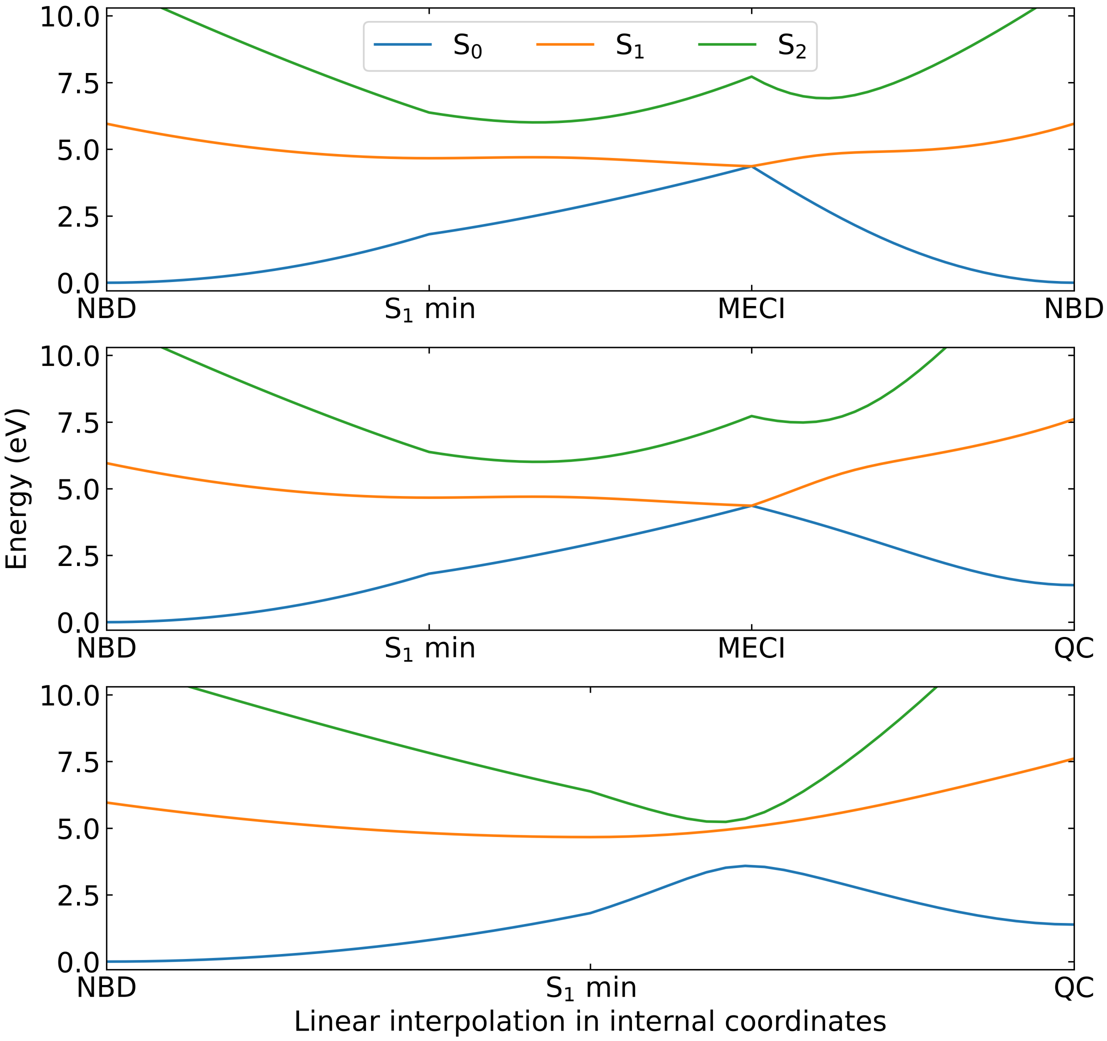

Figure S1: Potential energy cuts along linearly interpolated internal coordinates for prototypical pathways. Upper panel – trajectories reaching MECI and returning to NBD; middle – trajectories reaching MECI and isomerising to QC; bottom – trajectories converting directly into QC without reaching MECI. The S_1 minimum is passed through just before any bifurcation occurs and is thus included in all cuts.

Finally, the outlier parameter, γ, may be introduced to filter out points with insufficient density. The progressive increase in the number of outliers with γ is shown in Fig. S5 and the quality reported in Table S6.

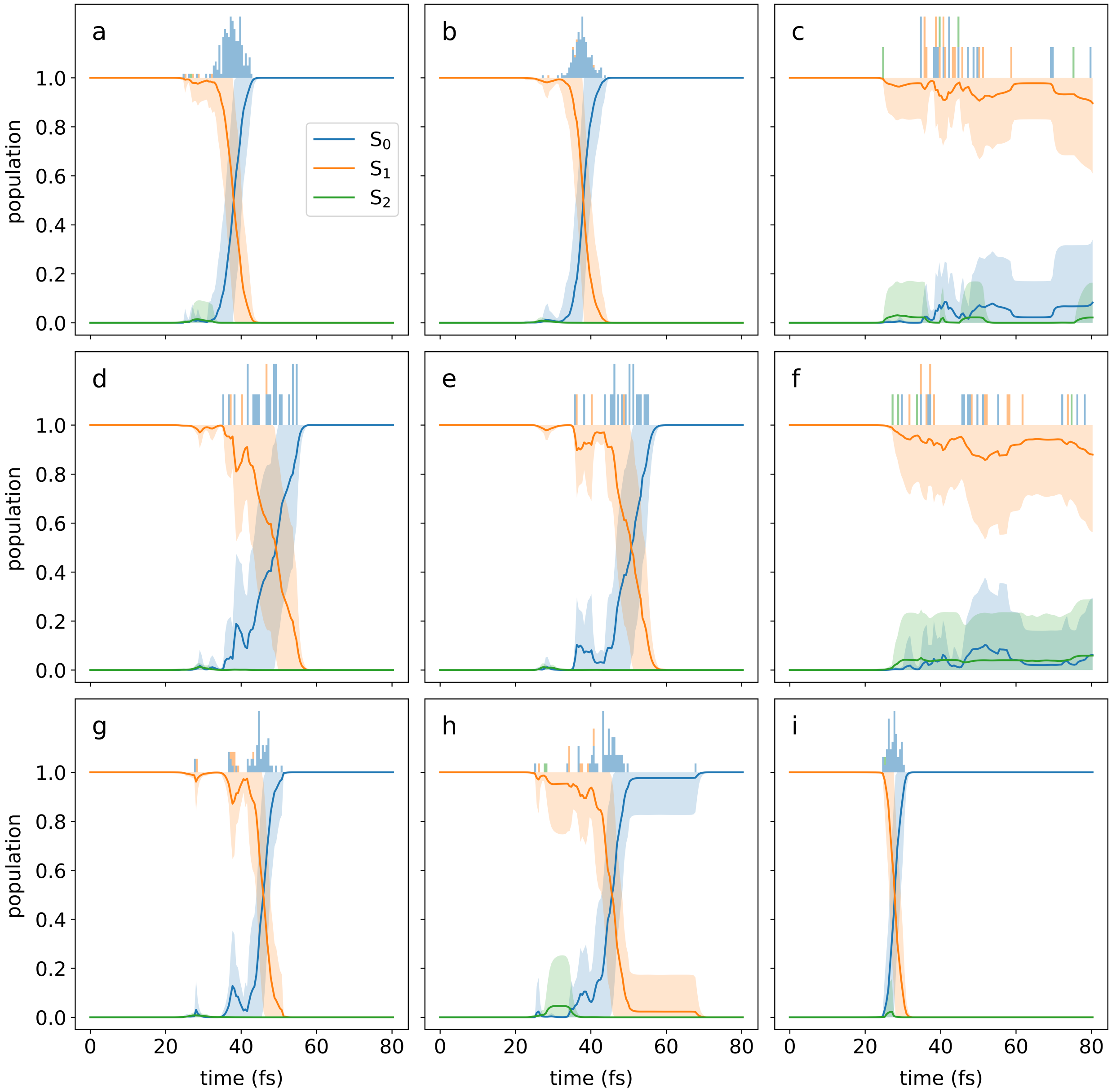

Figure S2: Population dynamics of the relaxation channels displayed in Fig. 8. Full lines show the average quantum populations for each pathway on each state and the shading captures one standard deviation (truncated to $[0, 1]$). Above the populations, histograms show active surface changes (hops), where the colours label the final state. The histograms are not normalised and not to scale between subplots.

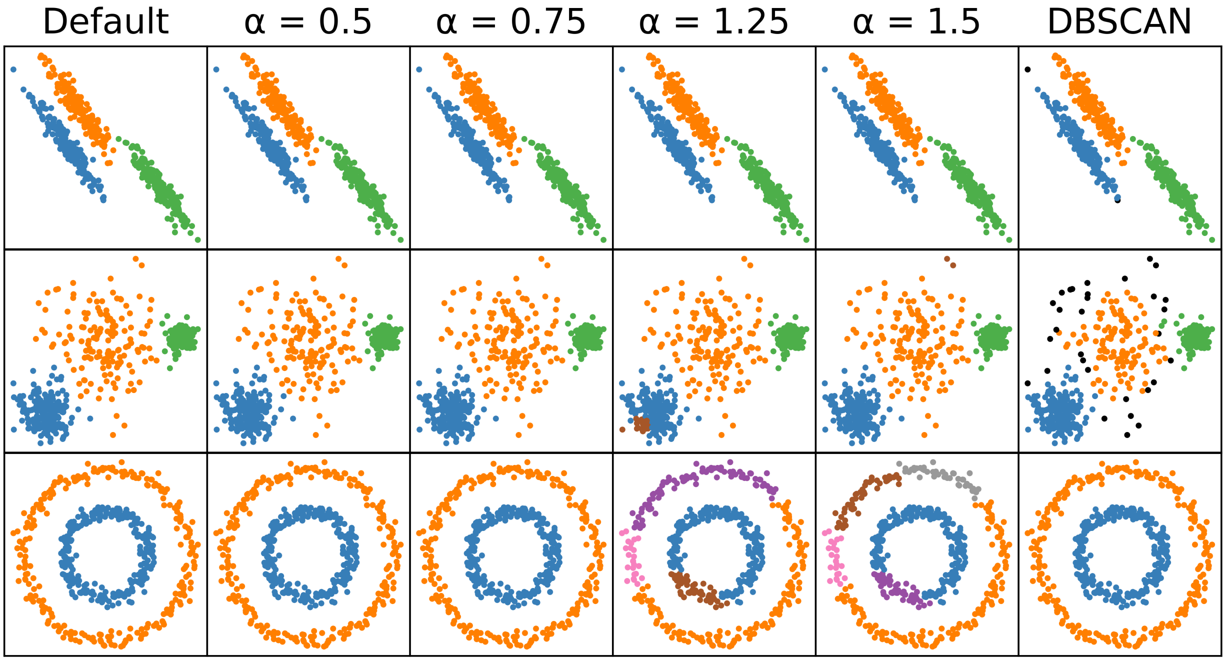

Figure S3: The results of clustering of DONKEY with selected α values algorithms; DBSCAN included for reference. The top row shows the results for the *Blobs* dataset, the middle row for the *Varied*, and the bottom row the *Circles*. The different algorithms are shown in each column, as labelled, with the new DONKEY algorithm in the first column. The identified clusters are assigned different colours (blue, orange, green, magenta, *etc*), with outlier points in black.

Table S4: Performance of the DONKEY on the synthetic 2D datasets with varying α as measured by homogeneity (first row), completeness (second row), and V-measure (third row).

	Default	$\alpha = 0.5$	$\alpha = 0.75$	$\alpha = 1.25$	$\alpha = 1.5$	DBSCAN
Blobs	**1.0000**	**1.0000**	**1.0000**	**1.0000**	**1.0000**	**1.0000**
	**1.0000**	**1.0000**	**1.0000**	**1.0000**	**1.0000**	0.9807
	**1.0000**	**1.0000**	**1.0000**	**1.0000**	**1.0000**	0.9902
Varied	0.8607	0.8607	0.8607	0.8607	0.8607	**0.8628**
	**0.8627**	**0.8627**	**0.8627**	**0.8627**	**0.8627**	0.7590
	**0.8617**	**0.8617**	**0.8617**	**0.8617**	**0.8617**	0.8076
Circles	**1.0000**	**1.0000**	**1.0000**	**1.0000**	**1.0000**	**1.0000**
	**1.0000**	**1.0000**	**1.0000**	0.6829	0.3501	**1.0000**
	**1.0000**	**1.0000**	**1.0000**	0.8116	0.5187	**1.0000**

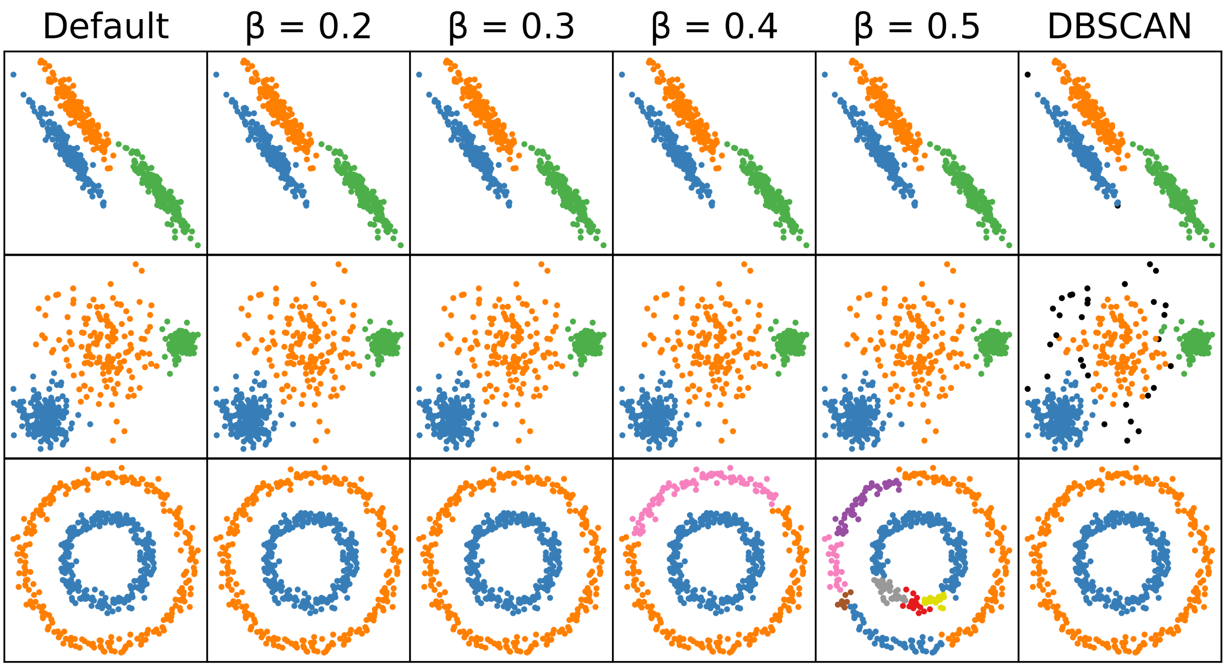

Figure S4: The results of clustering of DONKEY with selected β values algorithms; DBSCAN included for reference. The top row shows the results for the *Blobs* dataset, the middle row for the *Varied*, and the bottom row the *Circles*. The different algorithms are shown in each column, as labelled, with the new DONKEY algorithm in the first column. The identified clusters are assigned different colours (blue, orange, green, magenta, *etc*), with outlier points in black. Some colours are duplicated in Circles–$\beta = 0.5$ to the large number of detected clusters.

Table S5: Performance of the DONKEY on the synthetic 2D datasets with varying β as measured by homogeneity (first row), completeness (second row), and V-measure (third row).

	Default	$\beta = 0.2$	$\beta = 0.3$	$\beta = 0.4$	$\beta = 0.5$	DBSCAN
	**1.0000**	**1.0000**	**1.0000**	**1.0000**	**1.0000**	**1.0000**
Blobs	**1.0000**	**1.0000**	**1.0000**	**1.0000**	**1.0000**	0.9807
	**1.0000**	**1.0000**	**1.0000**	**1.0000**	**1.0000**	0.9902
	0.8607	**0.8715**	0.8607	0.8634	0.8607	0.8628
Varied	0.8627	**0.8738**	0.8627	0.7719	0.8463	0.7590
	0.8617	**0.8727**	0.8617	0.8151	0.8535	0.8076
	**1.0000**	**1.0000**	**1.0000**	**1.0000**	**1.0000**	**1.0000**
Circles	**1.0000**	**1.0000**	**1.0000**	0.4917	0.4533	**1.0000**
	**1.0000**	**1.0000**	**1.0000**	0.6592	0.6239	**1.0000**

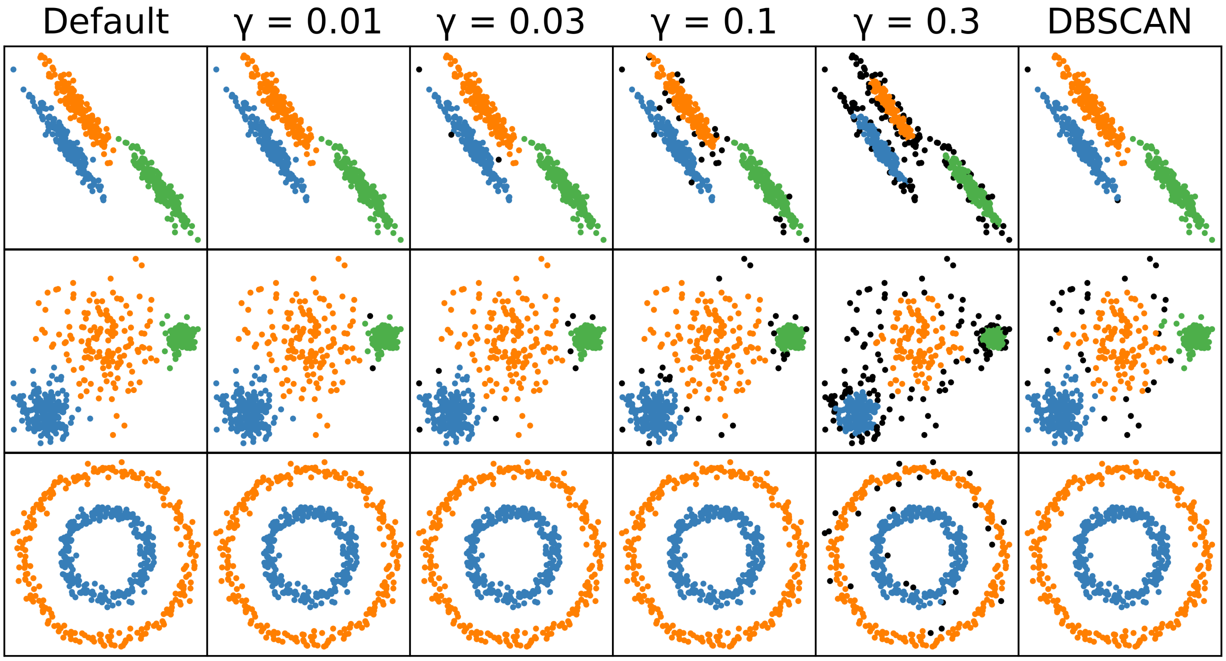

Figure S5: The results of clustering of DONKEY with selected γ values algorithms; DBSCAN included for reference. The top row shows the results for the *Blobs* dataset, the middle row for the *Varied*, and the bottom row the *Circles*. The different algorithms are shown in each column, as labelled, with the new DONKEY algorithm in the first column. The identified clusters are assigned different colours (blue, orange, green, magenta, *etc*), with outlier points in black.

Table S6: Performance of the DONKEY on the synthetic 2D datasets with varying γ as measured by homogeneity (first row), completeness (second row), and V-measure (third row).

	Default	$\gamma = 0.01$	$\gamma = 0.03$	$\gamma = 0.1$	$\gamma = 0.3$	DBSCAN
	**1.0000**	**1.0000**	**1.0000**	0.9525	0.7549	**1.0000**
Blobs	**1.0000**	**1.0000**	0.9734	0.8401	0.5993	0.9807
	**1.0000**	**1.0000**	0.9865	0.8928	0.6682	0.9902
	0.8607	0.8729	0.8962	**0.9033**	0.7378	0.8628
Varied	**0.8627**	0.8579	0.8436	0.7976	0.5870	0.7590
	0.8617	0.8653	**0.8691**	0.8472	0.6538	0.8076
	**1.0000**	**1.0000**	**1.0000**	**1.0000**	0.9602	**1.0000**
Circles	**1.0000**	**1.0000**	**1.0000**	**1.0000**	0.7770	**1.0000**
	**1.0000**	**1.0000**	**1.0000**	**1.0000**	0.8589	**1.0000**

References

(1) Pedregosa, F.; Varoquaux, G.; Gramfort, A.; Michel, V.; Thirion, B.; Grisel, O.; Blondel, M.; Prettenhofer, P.; Weiss, R.; Dubourg, V.; Vanderplas, J.; Passos, A.; Cournapeau, D.; Brucher, M.; Perrot, M.; Duchesnay, E. Scikit-learn: Machine Learning in Python. *Journal of Machine Learning Research* **2011**, *12*, 2825–2830.

(2) Gates, A. J.; Ahn, Y.-Y. The impact of random models on clustering similarity. *J. Mach. Learn. Res.* **2017**, *18*, 3049–3076.

(3) Rand, W. M. Objective criteria for the evaluation of clustering methods. *Journal of the American Statistical association* **1971**, *66*, 846–850.
